# Fucoidan from Fucus Vesiculosus Alleviates Interstitial Cystitis by Suppressing the AKT/NLRP3 Inflammasome Axis

**DOI:** 10.3390/ph19091427

**Published:** 2026-09-10

**Authors:** Chun-Shuo Hsu, Chu-Liang Lin, Yong-Syuan Chen, Hung-Ju Chien, Yi-Hsien Hsieh

**Affiliations:** 1 Institute of Medicine, Chung Shan Medical University, Taichung 40201, Taiwan; a123014@tzuchi.com.tw (C.-S.H.); like751019@gmail.com (C.-L.L.); 2 Department of Obstetrics and Gynecology, Dalin Tzu Chi Hospital, Buddhist Tzu Chi Medical Foundation, Chiayi 62247, Taiwan; 3 School of Medicine, Tzu Chi University, Hualien 970374, Taiwan; 4 Department of Medical Research, Chung Shan Medical University Hospital, Taichung 40201, Taiwan; cshe1470@csh.org.tw; 5 Department of Obstetrics and Gynecology, Changhua Christian Hospital, Changhua 50006, Taiwan

**Keywords:** fucoidan, interstitial cystitis, urothelium, AKT, NLRP3 inflammasome, TNFα, cyclophosphamide

## Abstract

**Background/Objectives:** Interstitial cystitis/bladder pain syndrome (IC/BPS) is a chronic inflammatory bladder disorder that lacks an effective treatment agent. Fucoidan (FUC), isolated from Fucus vesiculosus, is a sulfated polysaccharide that exhibits potent anti-inflammatory and anti-fibrotic activities. This study investigated the protective effects and underlying mechanisms of FUC in IC/BPS. **Methods:** NLRP3 inflammasome activation and AKT signaling were evaluated by Western blotting, qRT-PCR, immunofluorescence, pharmacological inhibition, gene silencing, and overexpression. Molecular docking assessed potential FUC–protein interactions. In vivo mouse model evaluated the protective effects and toxicity of FUC. **Results:** In SV-HUC-1 cells, FUC significantly attenuated TNF-α-induced expression of NLRP3 and cleaved caspase-1 and c-IL-1β expression at both the transcriptional and protein levels. Residual inflammasome activity after FUC treatment remained further suppressible by MCC950 or siNLRP3. FUC also reduced TNFα-induced AKT phosphorylation, whereas AKT silencing and overexpression enhanced and partially reversed the inhibitory effect of FUC on inflammasome signaling, respectively. The molecular docking results suggested potential interactions between FUC and key inflammasome-related proteins, including NLRP3, caspase-1, IL-1β, and AKT. In interstitial cystitis mice, FUC mitigated cyclophosphamide (CYP)-induced bladder injury, reduced bladder expression of inflammasome-associated markers, and exhibited no significant toxicity in major organs. **Conclusions:** These findings indicate that FUC alleviates urothelial inflammatory injury and experimental cystitis, at least in part, by suppressing the AKT/NLRP3 inflammasome axis, supporting its potential as a therapeutic candidate for IC/BPS.

## 1. Introduction

Interstitial cystitis/bladder pain syndrome (IC/BPS) is a chronic and debilitating bladder disorder characterized by persistent pelvic pain, pressure or discomfort associated with the bladder, and urinary urgency and frequency in the absence of overt infection or other identifiable causes [1]. Increasing evidence indicates that IC/BPS involves profound abnormalities in the bladder microenvironment, particularly within the urothelium, where epithelial dysfunction, barrier impairment, altered sensory signaling, and inflammatory activation are now regarded as central components of disease biology [2]. Because urothelial cells actively sense environmental stress and participate in down-stream inflammatory and nociceptive signaling, targeting urothelial inflammatory pathways has emerged as a rational therapeutic strategy for IC/BPS [2,3].

Accumulating evidence indicates that the NLRP3 inflammasome plays a critical functional role in the progression of IC/BPS [4,5]. Studies using LPS-induced IC models have demonstrated that curcumin effectively attenuates chronic inflammation-associated IC/BPS by modulating the NLRP3 inflammasome/IL-1β-related TGF-β/Smad signaling pathway [6]. Furthermore, dapansutrile has been shown to alleviate cyclophosphamide-induced interstitial cystitis [7], further supporting the pivotal role of the NLRP3 inflammasome pathway in IC/BPS. AKT signaling plays an important role in experimental cystitis and urothelial dysfunction [8]. Elevated AKT phosphorylation has been observed in cyclophosphamide (CYP)-induced bladder inflammation, while its inhibition improves bladder function [9]. Additionally, bladder epithelial tissues from IC/BPS patients exhibit aberrant AKT activation and dysregulated downstream signaling. The PI3K/AKT/mTOR pathway is also associated with inflammatory and injury responses in IC models [10]. However, whether AKT serves as a functional upstream mediator linking these signals to NLRP3 inflammasome activation in urothelial inflammation remains incompletely defined.

Fucoidan (FUC), a class of sulfated polysaccharides predominantly derived from brown seaweeds, has attracted substantial attention because of its anti-inflammatory, antioxidant, and immunomodulatory properties [11,12]. Importantly, FUC is not a single uniform molecule; its biological activity can vary according to source species, molecular weight, composition, and degree of sulfation [13,14]. Recent studies further suggest that FUC can regulate defined inflammatory signaling modules rather than merely exerting nonspecific cytokine suppression; for example, FUC has been reported to inhibit NLRP3 inflammasome activation in non-urologic disease models [15]. Nonetheless, the mechanistic potential of FUC in urothelial inflammation and IC/BPS remains poorly characterized. Therefore, we assessed the effects and molecular mechanism of FUC in IC/BPS in vitro and in vivo.

## 2. Results

### 2.1. FUC Suppresses TNFα-Induced NLRP3 Inflammasome Activation in SV-HUC-1 Cells

**Figure 1:**
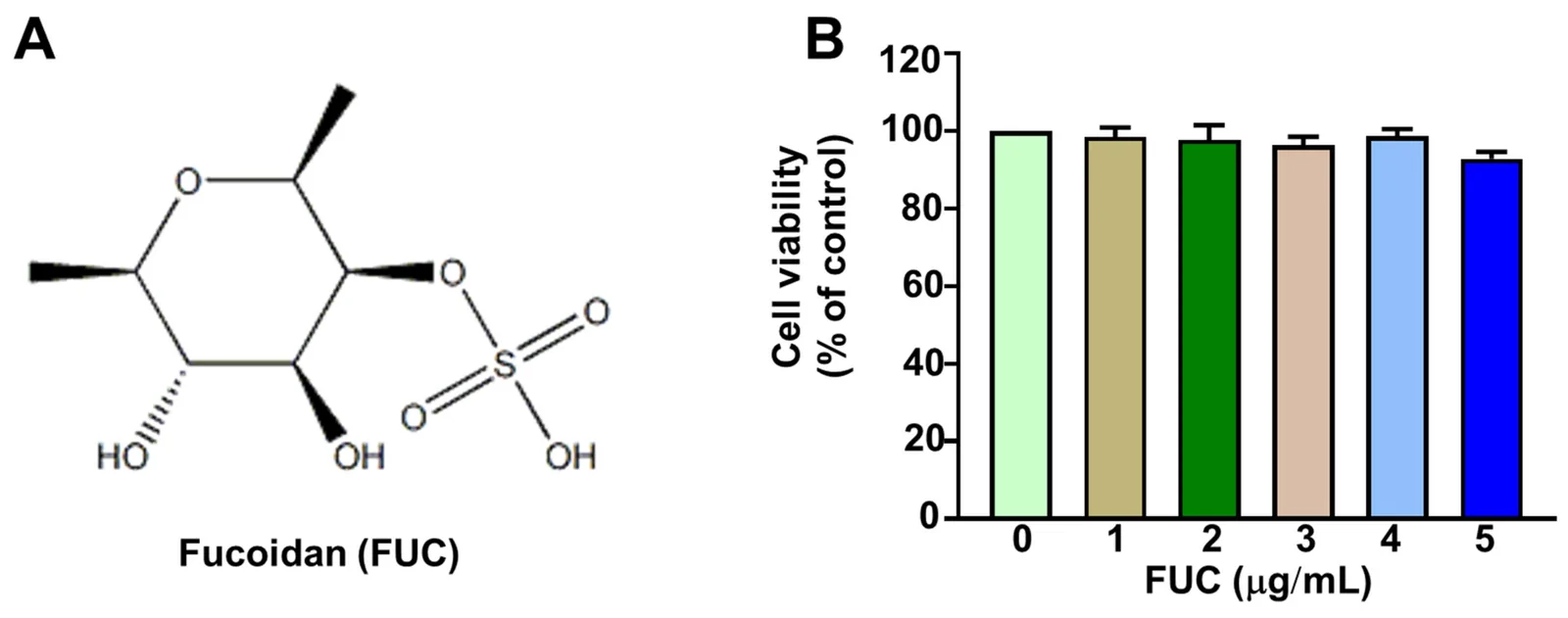
FUC preserves the viability of SV-HUC-1 cells. (**A**) Chemical structure of fucoidan (FUC), showing sulfated fucose-containing polysaccharides. (**B**) Cell viability of SV-HUC-1 normal human urinary tract epithelial cells after treatment with increasing concentrations of FUC ranging from 0 to 5 μg/mL. Data are presented as the mean ± SD from at least three independent experiments.

To investigate the mechanistic role of FUC in IC/BPS, we first evaluated the cytotoxicity profile of sulfated fucose-containing polysaccharides (Figure 1A) in normal SV-HUC-1 human urinary tract epithelial cells. Cell viability analysis showed that FUC did not induce substantial cytotoxicity across a concentration range of 0 to 5 μg/mL (Figure 1B).

**Figure 2:**
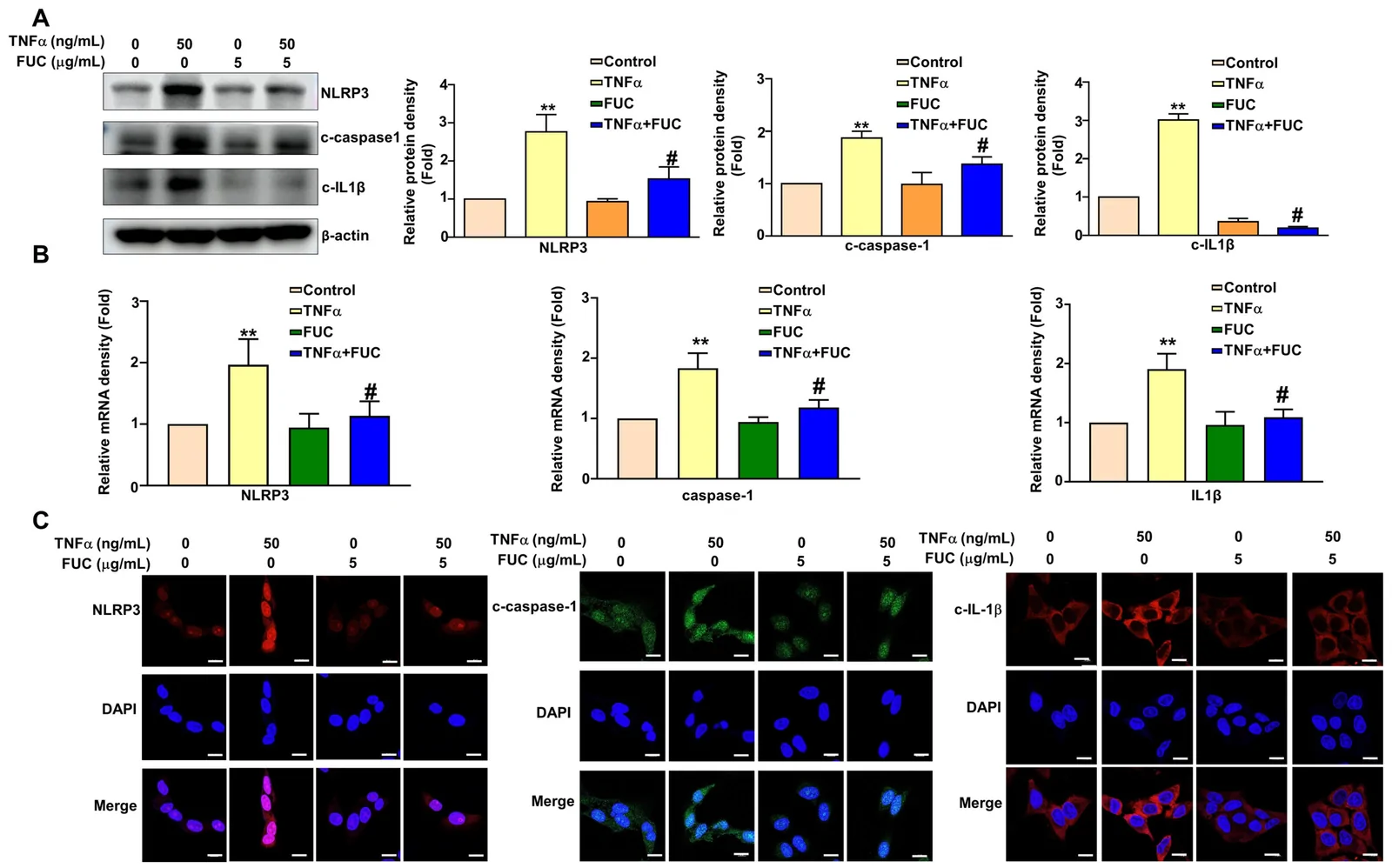
FUC suppresses TNFα-induced NLRP3 inflammasome activation in SV-HUC-1 cells. (**A**) SV-HUC-1 cells treated with TNFα, FUC, or TNFα+FUC were detected with protein expression of NLRP3, c-caspase-1, and c-IL-1β using Western blotting. Densitometric quantification of at least three independent Western blots was performed for each target. β-actin was used as the loading control. (**B**) Quantitative PCR was employed to determine mRNA expression of NLRP3, caspase-1, and IL-1β. GAPDH was used as the assay normalization control. (**C**) Immunofluorescence staining revealed endogenous levels of NLRP3, c-caspase-1, and c-IL-1β, respectively, with DAPI nuclear counterstaining. Data are presented as the mean ± SD from at least three independent experiments. ** *p* < 0.01 vs. control; # *p* < 0.05 vs. TNFα-treated cells. Scale bar = 20 μm.

FUC-treated SV-HUC-1 viability remained largely unchanged when compared to that of untreated control cells. Since TNFα is one of the major pro-inflammatory molecules that are markedly elevated in IC/BPS patients [16], a TNFα-induced SV-HUC-1 in vitro inflammation model was established to investigate the functional role of FUC in urothelial inflammation. Our results demonstrated that TNFα stimulation markedly activated the NLRP3 inflammasome pathway in SV-HUC-1 cells, as shown by coordinated increases in NLRP3, cleaved-caspase-1 (c-caspase-1) and cleaved-IL-1β (c-IL1β). Western blot analysis showed that TNFα significantly enhanced NLRP3 protein abundance relative to untreated control cells, accompanied by marked induction of c-caspase-1 and cL-1β (Figure 2A), suggesting that TNFα was sufficient to trigger canonical inflammasome-associated inflammatory signaling in SV-HUC-1 cells. Notably, FUC co-treatment significantly mitigated NLRP3-associated inflammasome activation under TNFα induction. In addition to NLRP3, FUC also significantly reduced c-caspase-1 and c-IL-1β protein levels that were enhanced by TNFα. Further, mRNA expression data were consistent with the protein findings. Transcriptional activation of the inflammasome-related genes NLRP3, caspase-1 and IL-1β enhanced by TNFα were significantly attenuated by FUC co-treatment (Figure 2B). FUC thus appeared to regulate the NLRP3 inflammasome at both transcriptional and post-translational levels. Moreover, immunofluorescence staining results of NLRP3, c-caspase-1, and c-IL-1β further supported this observation. The enhanced immunoreactivity signals from anti-NLRP3, c-caspase-1 and c-IL-1β by TNFα in SV-HUC-1 cells were greatly attenuated by FUC co-treatment (Figure 2C). Together, these data demonstrated that FUC suppresses TNFα-induced NLRP3 inflammasome activation in normal SV-HUC-1 cells.

### 2.2. NLRP3 Inhibition or Knockdown Further Suppresses Inflammasome Activity Beyond FUC Treatment

**Figure 3:**
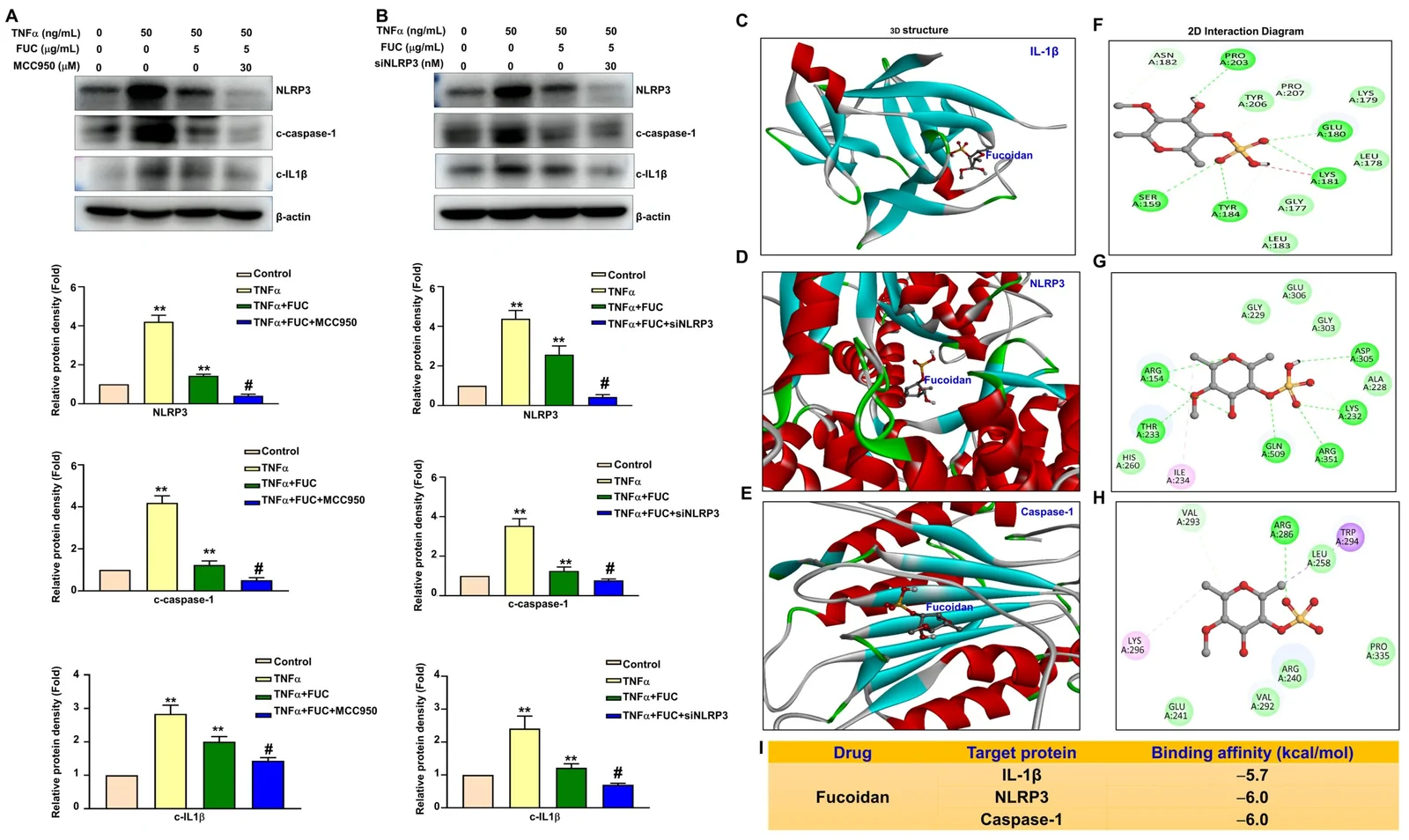
NLRP3 inhibition or knockdown further suppresses residual NLRP3 inflammasome activation after FUC treatment. SV-HUC-1 cells were treated with TNFα, TNFα plus FUC, or TNFα plus FUC and NLRP3 selective inhibitor MCC950. (**A**) Representative Western blots and quantitative analysis of NLRP3, c-caspase-1, and c-IL-1β. (**B**) Representative R and densitometric quantification was performed after siRNA-mediated NLRP3 knockdown. The three-dimensional docking models show the predicted binding interface for FUC at IL-1β (**C**), NLRP3 (**D**), and caspase-1 (**E**). (**F**–**H**) Two-dimensional ligand–protein interaction maps were derived from the corresponding docked complexes, illustrating the major predicted contact residues and interaction patterns between FUC and each target protein. (**I**) Predicted binding affinities of FUC for IL-1β, NLRP3, and caspase-1. Data are presented as the mean ± SD from at least three independent experiments. ** *p* < 0.01 vs. control; # *p* < 0.05 vs. TNFα+FUC-treated cells.

To extrapolate whether NLRP3 is a functionally relevant target within the anti-inflammatory activity of FUC, pharmacological inhibition and genetic knockdown approaches were next employed. This residual inflammatory activation provided a mechanistic rationale for employing a selective NLRP3 inhibitor, MCC950. Our results reveal that FUC attenuated TNFα-induced inflammasome responses and NLRP3, c-caspase-1, and c-IL1β levels. The use of MCC950 caused significant further suppression of NLRP3 inflammasome-associated protein expression in the TNFα+FUC-treated cells (Figure 3A). This NLRP3-dependent inflammasome pathway activation was reiterated by *NLRP3*-specific siRNA (siNLRP3) in TNFα+FUC-treated cells (Figure 3B). In line with pharmacologic inhibition of NLRP3 by MCC950, siNLRP3 significantly diminished residual inflammatory activation, supporting the hypothesis that NLRP3 is not merely a passive biomarker of TNFα-induced inflammation but a functional mediator of residual inflammasome response that persists after FUC treatment.

To substantiate the mechanistic correlation between FUC and the NLRP3 inflammasome cascade, molecular docking was conducted to uncover whether FUC interacted directly with the inflammasome markers. For IL-1β, the docked pose localized to a surface-accessible groove centered on a juxtaposition of the Lys179-Glu180-Lys181 region with adjacent Tyr184 that potentially provided a potent interface for sulfated polysaccharide FUC (Figure 3C), suggesting that electrostatic and hydrogen-bonding interactions might underlie the stabilization of the FUC/IL-1β complex (Figure 3F). For NLRP3, FUC occupied a broader central surface cleft in which Lys232, Asp305, Glu306, and Arg351 appeared to be the major interacting residues, with additional support from Gln509 (Figure 3D) that, together, comprise a predominantly polar/charge-assisted docking interface for FUC to interfere with NLRP3 conformational activation (Figure 3G). For caspase-1, the docked pose mapped to a region adjacent to the catalytic-core/interface area, with Arg240, Glu241, Arg286, Trp294, and Lys296 representing the most prominent interaction residues (Figure 3E). The coexistence of charged residues and the aromatic Trp294 suggested a mixed electrostatic and van der Waals interaction environment that could stabilize FUC binding at a functionally relevant protein surface (Figure 3H). The predicted binding energies were −5.7 kcal/mol for IL-1β and −6.0 kcal/mol for both NLRP3 and caspase-1 (Figure 3I), which indicates moderate affinities for FUC toward this inflammasome axis, suggesting that FUC may engage the NLRP3 inflammasome pathway at multiple molecular levels rather than acting on a single downstream target.

### 2.3. FUC Suppresses TNFα-Induced AKT Phosphorylation Upstream of NLRP3 Inflammasome Activation

**Figure 4:**
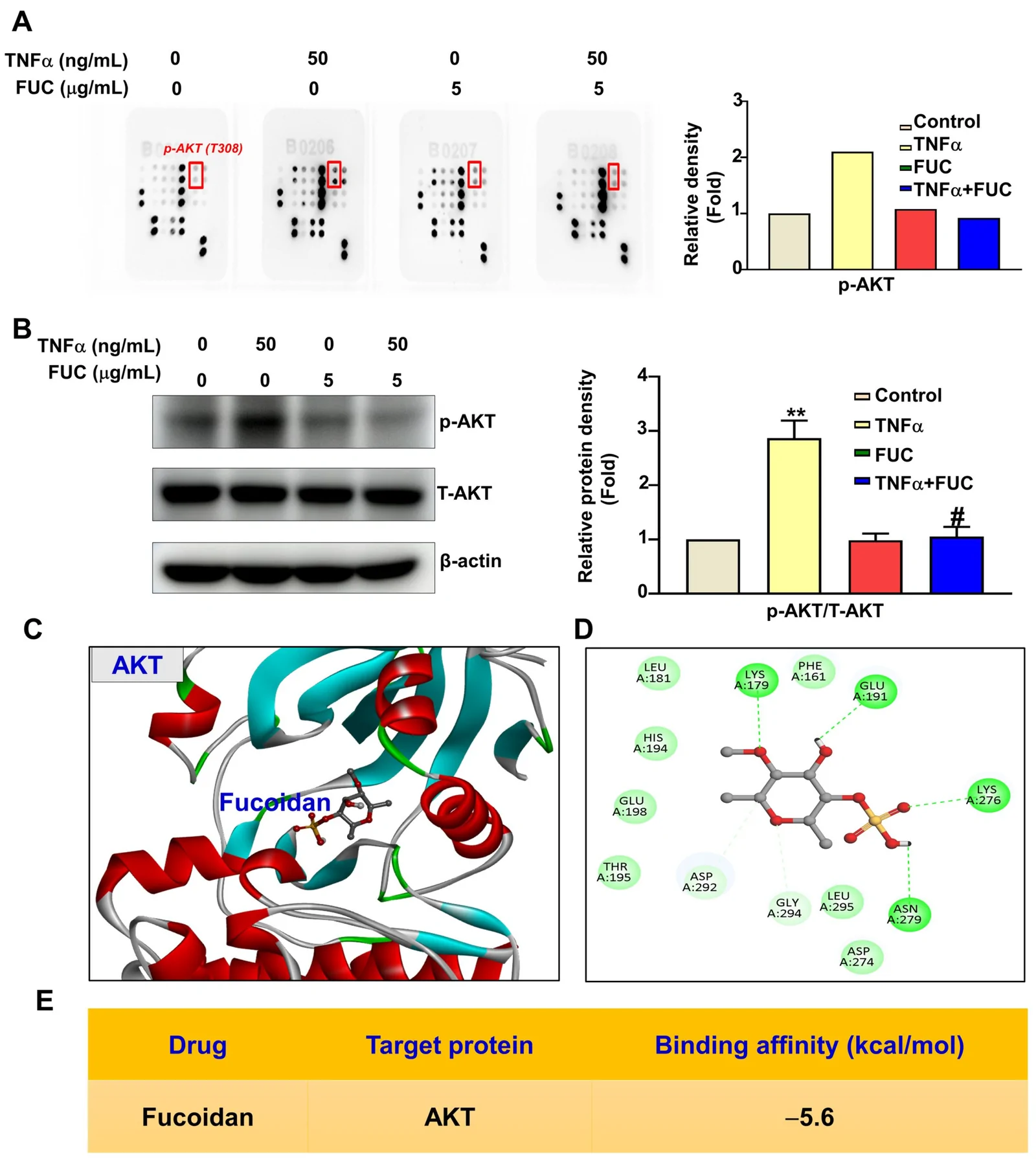
FUC inhibits TNFα-induced AKT phosphorylation in SV-HUC-1 cells. (**A**) Phospho-kinase array analysis was conducted using TNFα- and/or FUC-treated SV-HUC-1 cells. The boxed signals indicate p-AKT (T308), and the bar graph shows relative densitometric quantification. (**B**) Representative Western blots and quantification of p-AKT normalized to total AKT using β-actin as the loading control. (**C**) Three-dimensional molecular docking model showing the predicted binding pose of FUC within the AKT protein structure. (**D**) Two-dimensional ligand–protein interaction map derived from the docked AKT-FUC complex, illustrating the principal predicted contact residues and interaction pattern at the binding interface. (**E**) Predicted docking score/binding affinity of FUC for AKT. Data are presented as the mean ± SD from at least three independent experiments. ** *p* < 0.01 vs. control; # *p* < 0.05 vs. TNFα-treated cells.

Since kinase-mediated phosphorylation acts as a critical molecular switch to regulate NLRP3 inflammasome assembly, activation, and degradation [17], a phospho-kinase array was used to examine potent kinases that underline FUC-mediated NLRP3 inflammasome cascade activation. The data unveiled that phosphorylation of AKT at Thr308 was markedly enhanced by TNFα, whereas FUC co-treatment greatly reduced this TNFα-induced p-AKT signal (Figure 4A). Western blot validation confirmed that TNFα elevated the p-AKT/T-AKT ratio, while FUC reversely attenuated AKT phosphorylation without depleting total AKT (Figure 4B). These results imply that NLRP3 inflammasome inhibition by FUC was mediated by diminishing TNFα-induced AKT signaling activation rather than lowering the cellular abundance of AKT. Furthermore, molecular docking analysis demonstrated a docked pose placing FUC within a surface-accessible pocket of AKT, with the most prominent interactions centered on Lys179, Glu191, Lys276, and Asn279, with additional local support from nearby residues including Asp292 (Figure 4C,D), plausibly suggesting that sulfated polysaccharide FUC could be accommodated within the predominantly polar binding surface of AKT. This observation provides a consistent docking mode of FUC driven mainly by electrostatic and hydrogen bonding interactions. The predicted −5.6 kcal/mol binding affinity of FUC for AKT also indicates a moderately favorable interaction (Figure 4E). These findings support the hypothesis that FUC may act, at least in part, through direct engagement of AKT and consequent attenuation of signaling that leads to NLRP3 inflammasome activation.

### 2.4. AKT Expression Is Pivotal to FUC-Mediated Inhibition of the NLRP3 Inflammasome

**Figure 5:**
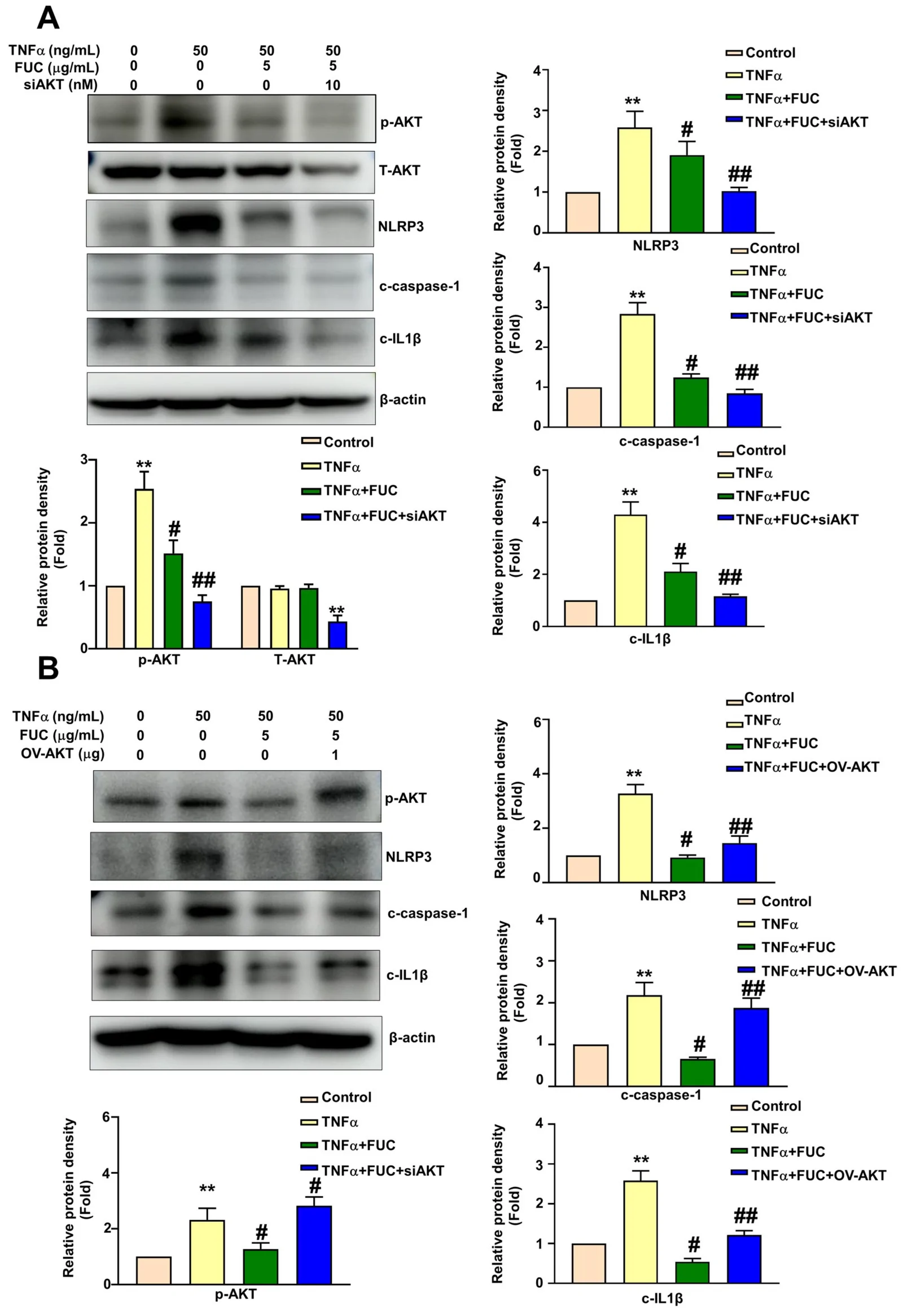
AKT functionally regulates FUC-mediated suppression of NLRP3 inflammasome activation. (**A**) Representative Western blots and densitometric quantification of p-AKT, T-AKT, NLRP3, c-caspase-1, and c-IL-1β after silencing of AKT (siAKT) in TNFα+FUC-treated SV-HUC-1 cells. (**B**) Representative Western blots and quantification of p-AKT, NLRP3, c-caspase-1, and c-IL-1β after AKT overexpression (OV-AKT) in TNFα+FUC-treated SV-HUC-1 cells. β-actin served as the loading control. Data are presented as the mean ± SD from at least three independent experiments. ** *p* < 0.01 vs. control; # *p* < 0.05 vs. TNFα-treated cells; ## *p* < 0.01 vs. the TNFα+FUC-treated cells.

To ascertain whether AKT is a pivotal signaling molecule that activates the NLRP3 inflammasome in this model, AKT was knocked down in TNFα/FUC-treated SV-HUC-1 cells. Our results show that the significantly reduced NLRP3 inflammasome activation was further mitigated when AKT was silenced, confirming that AKT knockdown suppresses inflammasome signaling beyond the mitigation effect achieved by FUC alone. The synergistic suppression of NLRP3, c-caspase-1, and c-IL-1β levels by co-treatments with FUC and siAKT potentially positions AKT upstream of the NLRP3 inflammasome axis in TNFα-stimulated SV-HUC-1 cells (Figure 5A). Moreover, the role of AKT was further examined in terms of ectopic AKT expression (OV-AKT). In Figure 5B, the FUC-lowered NLRP3, c-caspase-1, and c-IL-1β expression levels in TNFα-treated cells were significantly restored upon AKT overexpression, supporting the hypothesis that AKT activity plays a role in reversing the suppressive effect of FUC on inflammasome activation. These results suggested that FUC suppresses TNFα-stimulated NLRP3 inflammasome activation through an AKT-dependent mechanism.

### 2.5. FUC Alleviates Cyclophosphamide-Induced Bladder Inflammation In Vivo Without Overt Systemic Toxicity

**Figure 6:**
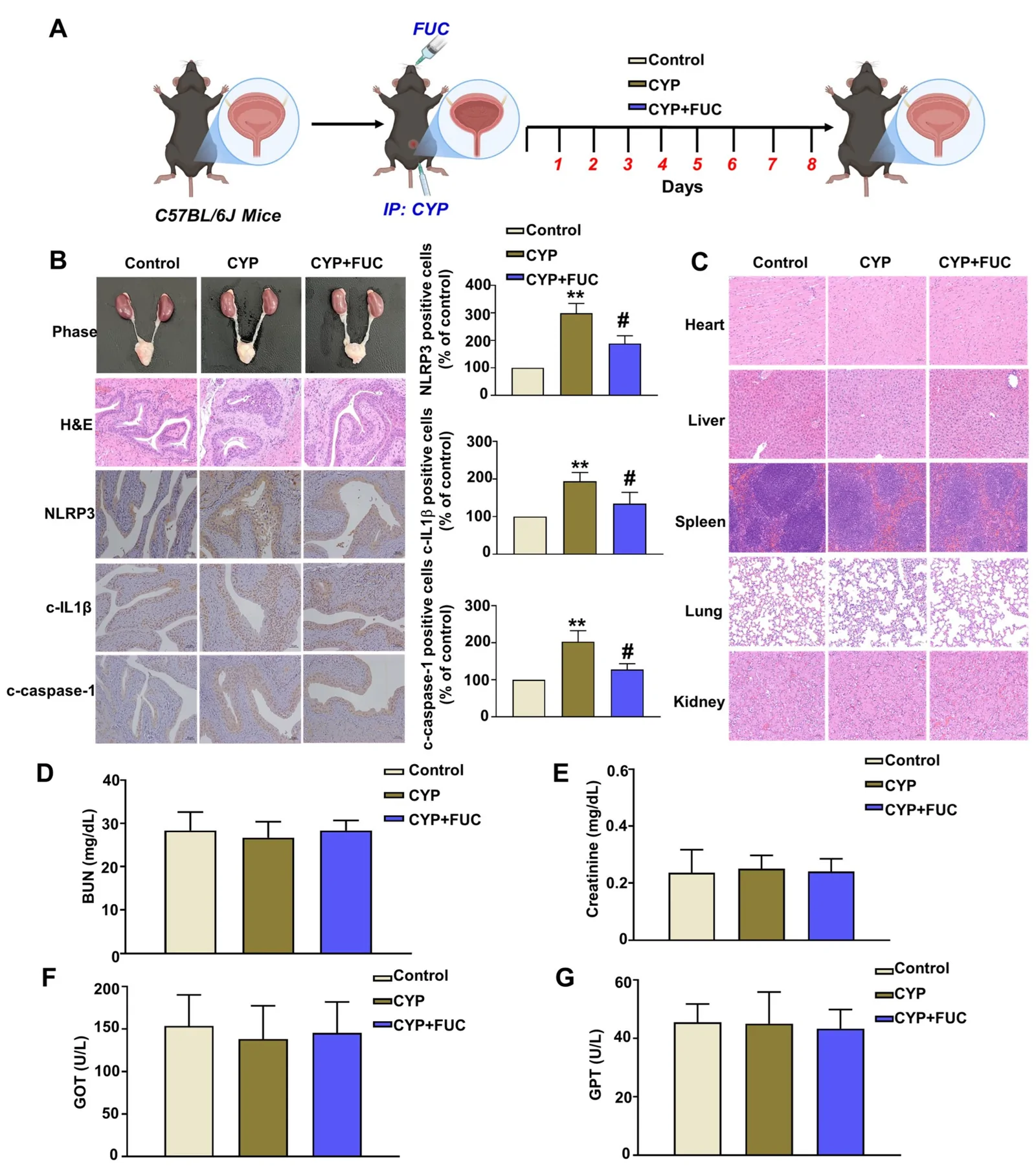
FUC mitigates cyclophosphamide-induced bladder inflammation in vivo without overt systemic toxicity. (**A**) Schematic of the in vivo experimental design using a mouse model of cyclophosphamide (CYP)-induced interstitial cystitis. (**B**) Gross bladder morphology, H&E staining, and immunohistochemical staining for NLRP3, c-IL-1β, and c-caspase-1 in bladder tissues, with corresponding quantitative analyses of IHC staining. (**C**) H&E staining of major organs, including the heart, liver, spleen, lung, and kidney, was performed to determine the absence of overt histopathological toxicity after FUC treatment. Serum blood urea nitrogen (**D**), creatinine (**E**), GOT (**F**), and GPT (**G**) levels were examined to detect renal or hepatic biochemical toxicity under the experimental conditions. Data are presented as the mean ± SD from at least three independent experiments. ** *p* < 0.01 vs. control; # *p* < 0.05 vs. TNFα-treated cells.

To corroborate FUC as a potent drug for future clinical use, the in vivo relevance of the AKT/NLRP3 mechanism was evaluated using a mouse model of cyclophosphamide (CYP)-induced IC. The experimental workflow involved CYP administration to induce bladder injury, followed by FUC treatment and tissue collection for histological and molecular analyses (Figure 6A). Gross examination demonstrated that CYP altered bladder morphology compared with control animals, whereas FUC treatment mitigated these macroscopic changes (Figure 6B). Immunohistochemical staining showed that CYP increased bladder expression of NLRP3, c-IL-1β, and c-caspase-1, while FUC treatment reduced the staining intensity of these inflammasome markers, consistent with the in vitro findings from SV-HUC-1 cells. These in vivo results suggest that FUC attenuated bladder mucosal inflammasome activation under CYP-induced inflammatory injury conditions. Potential systemic toxicity was also assessed. H&E staining of the heart, liver, spleen, lung, and kidney elicited no overt histopathological abnormalities attributable to FUC treatment (Figure 6C). Serum blood urea nitrogen (BUN) and creatinine levels remained statistically unchanged among groups, suggesting preserved renal function (Figure 6D,E). Similarly, treatment had no significant effect on body, and weight GOT and GPT levels did not show treatment-associated elevation that indicated no apparent hepatocellular toxicity under the experimental conditions (Figure 6F,G). Collectively, these data suggest that FUC mitigates CYP-induced bladder inflammation and suppresses NLRP3 inflammasome activation without detectable major organ, renal, or hepatic toxicity.

## 3. Discussion

**Figure 7:**
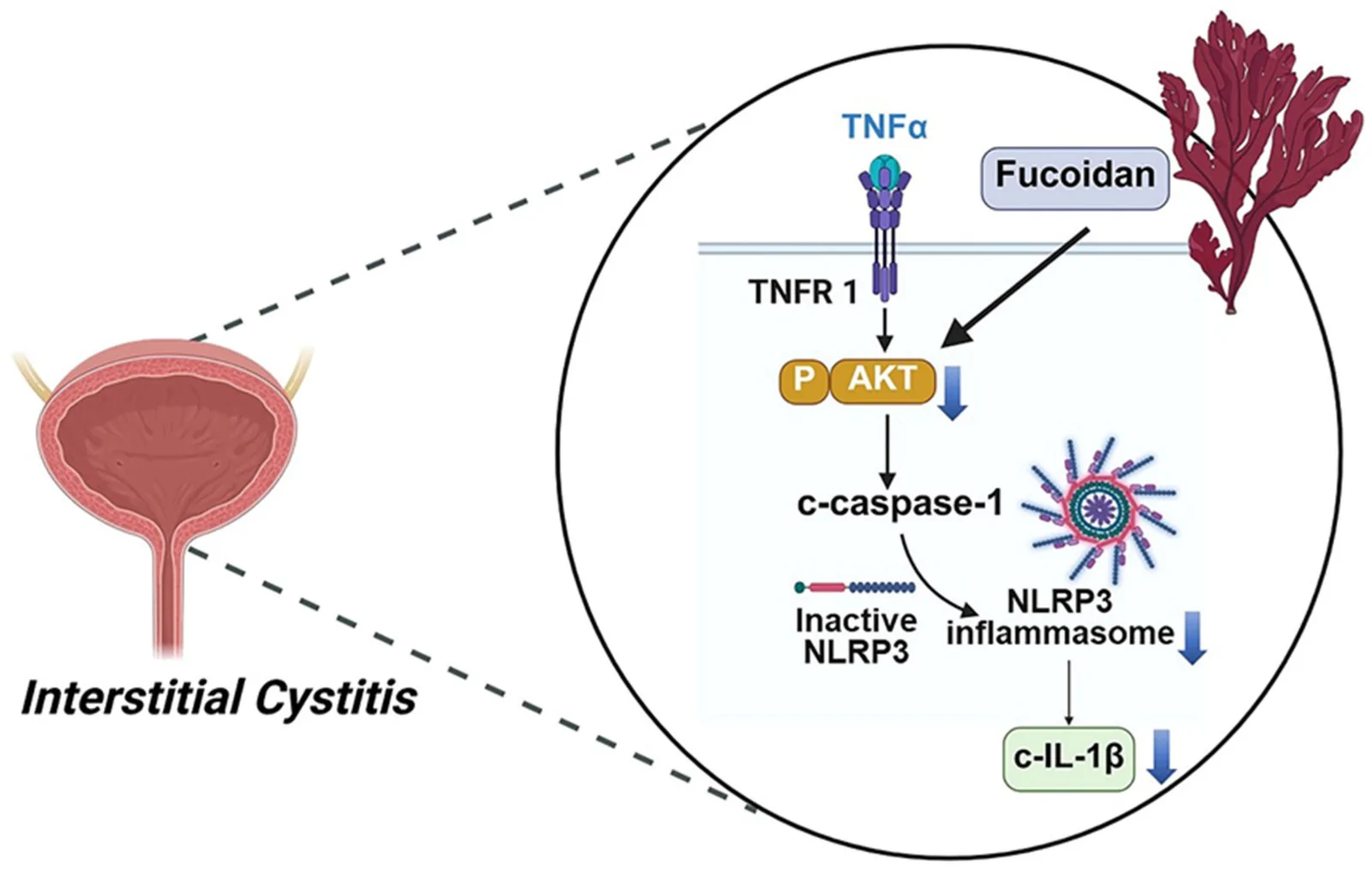
Mechanism by which FUC mitigates interstitial cystitis through inhibition of the AKT/NLRP3 inflammasome axis.

The present findings support a model in which FUC suppresses urothelial inflammatory signaling by attenuating AKT-dependent NLRP3 inflammasome activation. In TNFα-stimulated SV-HUC-1 cells, FUC reduced NLRP3 and cleaved caspase-1 and IL-1β at both the transcript and protein levels. These findings were reinforced by immunofluorescence and immunohistochemical analyses. At the pathway level, our data demonstrate that residual inflammasome activity after FUC treatment remained suppressible by MCC950 or siNLRP3, and TNFα-induced AKT phosphorylation was reduced by FUC and functionally linked to inflammasome output by AKT knockdown and rescue experiments. In vivo, the CYP-cystitis model extended this mechanism into bladder tissue, where FUC reduced pathological injury and inflammasome marker expression without evident renal, hepatic, or major organ toxicity. Together, these data showcase FUC as more than a generic anti-inflammatory compound and unveil a more defined urothelial signaling hierarchy centered on AKT and NLRP3 (Figure 7).

FUC suppresses TNFα-associated inflammatory signaling by reducing AKT phosphorylation, thereby inhibiting NLRP3 inflammasome activation, caspase-1 cleavage, and IL-1β maturation. In the cyclophosphamide-induced interstitial cystitis model, this mechanism is associated with reduced bladder inflammatory injury and preserved systemic safety profiles.

The mechanistic finding of the present study adds to the broadening view of IC/BPS as a disorder of persistent urothelial dysfunction coupled with chronic inflammatory signaling rather than a purely symptom-based syndrome [18]. Recent studies emphasize barrier disturbance, epithelial stress, altered immune crosstalk, and biomarker heterogeneity as central components of disease biology [19,20]. Within this context, TNFα is a rational experimental trigger as TNF-linked immune activity has been implicated in IC/BPS-related inflammation studies. Nonetheless, clinical trials evaluating the anti-TNFα agent adalimumab for the treatment of IC/BPS have shown it to be potentially effective at reducing symptoms in patients, but failed to demonstrate significant superiority over placebo in controlled studies [21,22]. This suggests that focusing on a sole upstream cytokine may be insufficient to manage such heterogenous and multifaceted pathogenesis. While TNFα is a broad marker of inflammation, the NLRP3 inflammasome is a specific intracellular multiprotein complex activated by cellular damage (DAMPs) and persistent pathogens (PAMPs) [4]. Hence, a major strength of the present data is that it specifically links FUC to the NLRP3/caspase-1/IL-1β axis. This finding is noteworthy because accumulating evidence from recent IC/BPS studies has increasingly implicated NLRP3 inflammasome activation as a disease-relevant mechanism rather than merely a secondary consequence of tissue injury [6,23]. In this regard, the current data position FUC within a growing class of interventions that can restrain NLRP3-related bladder inflammation. In this study, FUC reduced NLRP3 and cleaved caspase-1 cleaved IL-1β in TNFα-stimulated SV-HUC-1 cells, while pharmacologic or genetic disruption of NLRP3 further diminished the residual inflammatory activity remaining after FUC treatment. These findings indicate that NLRP3 is not merely a correlative marker within this system, but a functional mediator of the inflammatory phenotype that persists under TNFα stimulation. Notably, they also suggest that FUC does not simply suppress upstream cytokine signaling in a diffuse manner but restrains a defined inflammatory effector module linked to caspase-1 activation and IL-1β maturation.

The present data also extend the current understanding of kinase-dependent regulation of urothelial inflammasome activation by identifying AKT as a functionally relevant upstream mediator. Phosphorylation-dependent control has emerged as an important regulatory layer in NLRP3 inflammasome activation and turnover [17]. In parallel, AKT signaling has been implicated in experimental cystitis and in abnormal epithelial signaling states associated with IC/BPS [9,24]. In the present study, phospho-kinase array analysis first identified AKT phosphorylation as FUC-sensitive in NLRP3-mediated inflammasome signaling. More importantly, siAKT further attenuated NLRP3/caspase-1/IL-1β signaling, whereas AKT overexpression significantly restored this phenotype. The reciprocal behavior of these perturbation experiments supports a pathway model in which AKT contributes to maintaining TNFα-driven urothelial inflammasome signaling and represents an important upstream target of FUC. The role of AKT in bladder biology, however, is likely to be context-dependent. Although increased AKT activity has been associated with inflammatory dysfunction in CYP-induced cystitis and abnormal epithelial signaling [8], AKT-related pathways may also participate in adaptive or reparative responses depending on stimulus type, cellular compartment, and disease stage [25]. The present study’s findings thus support the conclusion that, under TNFα-driven urothelial inflammatory conditions, AKT contributes to NLRP3 inflammasome activation and is a potent target of FUC-mediated suppression.

The results of the molecular docking analyses further support the plausibility that FUC may engage the AKT/NLRP3 inflammasome axis at multiple molecular levels. Moderate predicted binding affinities were observed for IL-1β, NLRP3, caspase-1, and AKT, and the identified binding interfaces were chemically consistent with a sulfated polysaccharide ligand. These findings are consistent with previous studies showing that FUC attenuates inflammatory injury accompanied by reduced NLRP3 inflammasome activation in non-urologic disease models [26]. Furthermore, FUC is not a chemically uniform entity. Source species, extraction method, molecular weight distribution, sulfation pattern, and monosaccharide composition can materially influence biological activity [27]. Indeed, studies indicate that FUC can activate, rather than suppress, AKT-dependent or pro-inflammatory outputs in selected cellular contexts [28,29,30]. This heterogeneity is highly relevant to the present study, as it also underscores the need for rigorous structural characterization and structure–activity analyses in future translational work. In addition, while molecular docking provides a structurally informed rationale for potential protein–ligand interactions, it cannot distinguish direct binding from indirect pathway modulation. In future studies, we plan to employ the Cellular Thermal Shift Assay (CETSA) to evaluate the potential direct interaction of FUC with AKT and/or NLRP3 inflammasome-associated proteins in intact cells by assessing ligand-induced changes in protein thermal stability. These experiments will provide additional evidence to validate the docking predictions and clarify whether the observed modulation of the AKT/NLRP3 inflammasome pathway results from direct target engagement or indirect regulatory mechanisms.

Although our histological and molecular findings suggest the protective and anti-inflammatory effects of FUC in the CYP-induced bladder injury mice model, they lack functional outcomes directly relevant to IC/BPS. Future in vivo animal studies incorporating cystometric analysis and voiding stain paper (VSP) assessments will be required to determine whether the histological and molecular improvements induced by FUC translate into meaningful functional benefits.

## 4. Materials and Methods

### 4.1. Reagents and Antibodies

The fucoidan (FUC) was a commercially available preparation purchased from Sigma-Aldrich (St. Louis, MO, USA; Cat. No. F8190; CAS No. 9072-19-9). This FUC is derived from the brown alga *Fucus vesiculosus* and has an assay of ≥95%. FUC was dissolved in sterile water and stored at −80 °C until use. Recombinant human TNFα was obtained from CROYEZ (Taichung, Taiwan; catalog no. C01047) and reconstituted according to the manufacturer’s instructions. MCC950 was purchased from MedChemExpress (Monmouth Junction, NJ, USA; catalog no. HY-12815) and dissolved in DMSO prior to use. Cyclophosphamide monohydrate was purchased from Sigma-Aldrich (St. Louis, MO, USA; catalog no. C0768) and freshly prepared in sterile saline prior to animal administration. Fetal bovine serum (FBS; 35-010-cv) was purchased from Corning Life Sciences (Tewksbury, MA, USA). Full information for antibodies against NLRP3, c-caspase-1, c-IL-1β, phospho-AKT (Thr308), total-AKT and β-actin, together with horseradish peroxidase (HRP)-conjugated or fluorescent secondary antibodies, is provided in Supplementary Table S1.

### 4.2. Cell Culture and Treatments

SV-HUC-1 cells used as a non-malignant human urinary tract epithelial model in this study were purchased from Bioresource Collection and Research Center (BCRC-60358) and cultured in Ham’s F12 medium (Gibco, Thermo Fisher Scientific, Waltham, MA, USA; catalog no. 21127022) supplemented with 7% FBS at 37 °C in 5% CO_2_ incubator (ID370; Forma Steri-Cycle CO_2_ Incubators, Thermo Fisher Scientific, Waltham, MA, USA).

For FUC-treatment experiments, SV-HUC-1 cells were pretreated with FUC (5 μg/mL) for 2 h then added to the TNF-α (50 ng/mL) stimulation for 22 h. Following FUC pretreatment, FUC was not removed from the culture medium, and TNF-α was added directly to the FUC-containing medium. For molecular mechanism studies involving MCC950, SV-HUC-1 cells were pretreated with MCC950 (30 μM) for 2 h. MCC950 was not removed before TNF-α stimulation; instead, FUC+TNF-α were added directly to the MCC950-containing medium, and the cells were co-incubated with MCC950 and TNF-α+FUC for an additional 22 h.

### 4.3. Cell Viability Assay

Cell viability was assessed to identify a non-cytotoxic working concentration of FUC. SV-HUC-1 cells were seeded into 96-well plates at 5 × 10^4^ cells/well and allowed to adhere overnight. Cells were then treated with FUC at 0, 1, 2, 3, 4, and 5 μg/mL for 24 h. Viability was measured using MTT assay (GoldBio, St. Louis, MO, USA; catalog no. M-200) according to the manufacturer’s instructions. Absorbance was recorded at 570 nm using a BioTek microplate ELISA reader (ChromTech, Apple Valley, MN, USA). Cell viability was expressed as a percentage of untreated control cells.

### 4.4. siRNA-Mediated Knockdown and AKT Overexpression

For transient gene silencing, SV-HUC-1 cells (4 × 10^5^ cells) were transfected with siRNA specifically targeting NLRP3 or AKT, or with a non-targeting control siRNA, using Lipofectamine RNAiMAX transfection reagent (Invitrogen, Thermo Fisher Scientific, Waltham, MA, USA; catalog no. 13778150). Cells were used for downstream experiments 24–48 h after transfection, followed by FUC/TNFα treatment as described above. For AKT overexpression (Ov-AKT; 1 μg), cells were transfected with an AKT expression plasmid or corresponding empty vector control using Lipofectamine 3000 (Thermo Fisher Scientific Inc., Waltham, MA, USA). At 24 h after transfection, the cells were treated with FUC and TNF-α for an additional 24 h before subsequent functional and molecular analyses.

### 4.5. Western Blot

Protein lysates were extracted from SV-HUC-1 cells following different treatments for 24 h. Equal amounts of protein were separated by 8–12% SDS-polyacrylamide gel electrophoresis (SDS-PAGE) and subsequently transferred onto PVDF membranes. The membranes were incubated overnight at 4 °C with primary antibodies against NLRP3 (1:1000), cleaved-caspase-1 (1:1000), cleaved-IL-1β (1:1000), phospho-AKT (1:1000), total-AKT (1:2000), and β-actin (1:1000). After washing, the membranes were incubated with the appropriate horseradish peroxidase-conjugated secondary antibodies for 1 h at room temperature. Protein bands were visualized using Immobilon Western HRP Substrate (Millipore, Billerica, MA, USA) and detected with a chemiluminescence imaging system (ImageQuant LAS 4000 Mini, GE Healthcare, Chicago, IL, USA). Band intensities were quantified using ImageJ software (ImageJ 1.54p) and normalized to β-actin expression.

### 4.6. qRT-PCR Assay

Total RNA was extracted from FUC-treated SV-HUC-1 cells using TRIzol reagent, and RNA concentration and purity were assessed using a NanoDrop spectrophotometer (Thermo Fisher Scientific, Waltham, MA, USA). Subsequently, 1 μg of total RNA was reverse-transcribed into cDNA using GoScript™ Reverse Transcription Mix (Promega, Madison, WI, USA). Quantitative real-time PCR was performed using a StepOnePlus real-time PCR system (Applied Biosystems, Foster City, CA, USA). Relative mRNA expression levels of NLRP3, caspase-1, and IL-1β were calculated using the 2^−ΔΔCt^ method, with GAPDH used as the internal reference gene for normalization. The primer sequences were as follows: NLRP3, forward, 5′-GATCTTCGCTGCGATCAACAG-3′; reverse-5′-CGTGCATTATCTGAACCCCAC-3′; CASP1; forward-5′-GCACAAGACCTCTGACAGCA-3′; reverse-5′-TTGGGCAGTTCTTGGTATTC-3′; IL-1β, forward-5′-CCACAGACCTTCCAGGAGAATG-3′; reverse-5′- GTGCAGTTCAGTGATCGTACAGG-3′ and GAPDH-forward-5′-CATCATCCCTGCCTCTACTG-3′ and reverse-5′-GCCTGCTTCACCACCTTC-3′.

### 4.7. Immunofluorescence Staining

SV-HUC-1 cells were seeded on 8-well Lab-Tek Chambered Coverglass and treated with FUC for 24 h, then fixed with 4% PFA and blocked with 5% BSA for 2 h using the antibodies for NLRP3, c-caspase-1 or c-IL-1β, followed by incubation with fluorophore-conjugated secondary antibodies for 1 h in the dark. Nuclei were counterstained with DAPI reagent for 2 h. Immunofluorescence images were acquired using a ZEISS LSM 900 confocal microscope (Carl Zeiss, Oberkochen, Germany).

### 4.8. Human Phospho-Kinase Array

SV-HUC-1 cell lysates were treated with FUC for 24 h and analyzed using a Proteome Profiler Human Phospho-Kinase Array Kit (Catalog # ARY003C, R&D Systems, Minneapolis, MN, USA). Spot intensity was created using an ImageJ software and normalized to the internal positive-control spots on each membrane.

### 4.9. Molecular Docking

In silico molecular docking was performed using PyRx (version 0.8). The three-dimensional structure of FUC was obtained from PubChem (CID: 129532628) and energy-minimized using the UFF force field. Crystal or cryo-EM structures of human IL-1β, NLRP3, caspase-1, and AKT were retrieved from the RCSB Protein Data Bank or AlphaFold database with the following accession codes: IL-1β (PDB: 5R8E), NLRP3 (AF-Q96P20-F1-model_v6), caspase-1 (AF-P29466-F1-model_v6), and AKT (PDB: 4GV1). In order to dock, protein structures were prepared by removing water molecules and co-crystallized ligands where appropriate, followed by the addition of polar hydrogens. Docking grids were defined to encompass the entire protein surface. The exhaustiveness parameter was set to 256 to ensure thorough conformational sampling. The top-ranked binding poses were selected based on binding affinity (kcal/mol) and further analyzed using Discovery Studio Visualizer. Protein–ligand interactions, including hydrogen bonds, π-interactions, and hydrophobic contacts, were characterized using Discovery Studio 2025 Client.

### 4.10. IC Mouse Model and FUC Administration

Animal experiments conducted in this study were approved by the Institutional Animal Care and Use Committee at Chung Shan Medical University (IACUC number: 113142). Five-week-old female C57BL/6J mice were randomly allocated to the experimental groups using a randomization procedure (*n* = 5 per group). FUC was administered at 100 mg/kg by oral gavage once daily for 8 consecutive days concurrently with CYP exposure (*n* = 5 per group). Control animals received equivalent volumes of vehicle. Animals were monitored daily for general appearance, activity and body-weight changes. At the experimental endpoint following FUC administration, mice were anesthetized with anesthetic agent and dose and subsequently euthanized by CO_2_ inhalation.

### 4.11. Tissue Collection, Gross Morphology, H&E Staining and Immunohistochemistry

Bladder, heart, liver, spleen, lung and kidney tissues were fixed in 10% neutral buffered formalin, dehydrated, embedded in paraffin, and sectioned at 4–5 μm thickness. For hematoxylin and eosin (H&E) staining, tissue sections were stained with hematoxylin, differentiated, blued, counterstained with eosin, dehydrated and mounted. Histological images were captured using brightfield microscope. For immunohistochemistry (IHC), paraffin sections were deparaffinized and rehydrated. Tissues sections were incubated overnight at 4 °C with primary antibodies against NLRP3, c-caspase-1 and c-IL-1β, followed by HRP-conjugated secondary antibodies and DAB chromogen development. Nuclei were counterstained with hematoxylin. Stained sections were imaged using identical acquisition parameters across groups.

### 4.12. Serum Biochemical Analysis

Whole blood was centrifuged at 3000× *g* for 10 min to obtain serum. Serum levels of blood urea nitrogen (BUN), creatinine, glutamic oxaloacetic transaminase (GOT), and glutamic pyruvic transaminase (GPT) were measured using the Comprehensive II Panel and analyzed with the ENNOLIFE Clinical Chemistry Analyzer (ProtectLife International Biomedical Inc., Taipei, Taiwan).

### 4.13. Statistical Analysis

In vitro results of data are expressed as mean ± standard deviation (SD) from three independent experiments. For the in vivo animal experiments, five mice were included in each experimental group (*n* = 5 per group), and each animal was considered an independent biological replicate. Mice were randomly allocated to the experimental groups. Statistical analyses were performed using GraphPad Prism 6.0. Comparisons between two groups were conducted using a two-tailed Student’s *t*-test, whereas comparisons among multiple groups were performed using one-way ANOVA followed by Tukey’s post hoc test. A *p*-value < 0.05 or <0.01 was considered statistically significant.

## 5. Conclusions

The present study identifies FUC as a promising urothelial anti-inflammatory agent that mitigates TNFα-driven inflammatory injury and experimental cystitis via modulation of the AKT/NLRP3 inflammasome axis. These findings establish a coherent mechanistic framework for future investigations to validate direct target engagement, further characterize NLRP3 inflammasome outputs, and evaluate whether structurally defined FUC preparations can be developed into effective therapeutic strategies for IC/BPS.

## Data Availability

The original contributions presented in this study are included in the Supplementary Material. Further inquiries can be directed to the corresponding authors.

## Supplementary Materials

The following supporting information can be downloaded at: https://www.mdpi.com/article/10.3390/ph19091427/s1, Table S1: Antibodies used in this study.

## Abbreviations

The following abbreviations are used in this manuscript:

AKT	AKT serine/threonine kinase
ALT	alanine aminotransferase
AST	aspartate aminotransferase
BUN	blood urea nitrogen
c-IL-1β	cleaved interleukin-1 beta
CYP	cyclophosphamide
DAPI	3,3′-diaminobenzidine (DAB); 4′,6-diamidino-2-phenylindole
FBS	fetal bovine serum
FUC	fucoidan
GOT	glutamic oxaloacetic transaminase
GPT	glutamic pyruvic transaminase
H&E	hematoxylin and eosin
IF	immunofluorescence
IHC	immunohistochemistry
IC/BPS	interstitial cystitis/bladder pain syndrome
IL-1β	interleukin-1 beta (IL-1β)
NLRP3	nucleotide-binding oligomerization domain, leucine-rich repeat and pyrin domain-containing 3 (NLRP3)
PBS	phosphate-buffered saline (PBS)
PDB	Protein Data Bank (PDB)
qPCR	quantitative polymerase chain reaction (qPCR)
siRNA	small interfering RNA
SV-HUC-1	SV40-immortalized human uroepithelial cells-1
TNFα	tumor necrosis factor-alpha
