# Supplementary material for: Fucoidan from Fucus Vesiculosus Alleviates Interstitial Cystitis by Suppressing the AKT/NLRP3 Inflammasome Axis
Source: Pharmaceuticals (Basel). 2026 Sep 10;19(9):1427. doi: 10.3390/ph19091427 (PMC13610977; doi:10.3390/ph19091427)
Supplement: Supplementary file 1 [file pharmaceuticals-19-01427-s001.zip › pharmaceuticals-4519944-supplementary.pdf]

Supplementary Table S1. Antibodies used in this study

**Western blotting**			
**Target protein**	**Cat. No.**	**Company**	**Dilute factor**
NLRP3	15101S	Cell signaling	1:1000
Cleaved-caspase-1	sc-56036	Santa Cruz	1:1000
Cleaved-IL-1β	ab283818	Abcam	1:1000
p-AKT (Thr308)	sc-271966	Santa Cruz	1:1000
AKT	sc-56878	Santa Cruz	1:1000
β-actin	sc-47778	Santa Cruz	1:10000
HRP-conjugated secondary antibody, anti-rabbit IgG	111-035-003	Jackson ImmunoResearch	1:5000
HRP-conjugated secondary antibody, anti-mouse IgG	115-035-003	Jackson ImmunoResearch	1:5000
**Immunofluorescence staining**			
NLRP3	sc-518123	Santa Cruz	1:50
Cleaved-caspase-1	sc-392736	Santa Cruz	1:50
Cleaved-IL-1β	IR114-473	iREAL	1:200
Fluorophore-conjugated secondary antibody, anti-rabbit IgG	A11011	Invitrogen	1:500
Fluorophore-conjugated secondary antibody, anti-mouse IgG	115-545-003	Jackson ImmunoResearch	1:250
**Immunohistochemistry**			
NLRP3	GTX133569	Genetex	1:100
Cleaved-caspase-1	AF4005	Affinity	1:100
Cleaved-IL-1β	AF4006	Affinity	1:100
